# A possible role for mitochondrial-derived peptides humanin and MOTS-c in patients with Q fever fatigue syndrome and chronic fatigue syndrome

**DOI:** 10.1186/s12967-019-1906-3

**Published:** 2019-05-14

**Authors:** Ruud P. H. Raijmakers, Anne F. M. Jansen, Stephan P. Keijmel, Rob ter Horst, Megan E. Roerink, Boris Novakovic, Leo A. B. Joosten, Jos W. M. van der Meer, Mihai G. Netea, Chantal P. Bleeker-Rovers

**Affiliations:** 1 Radboud Expertise Center for Q Fever, Department of Internal Medicine, Division of Infectious Diseases 463, Radboud University Medical Center, P.O. Box 9101, 6500 HB Nijmegen, The Netherlands; 2 Department of Internal Medicine, Radboud University Medical Center, P.O. Box 9101, 6500 HB Nijmegen, The Netherlands; 3 Murdoch Children’s Research Institute, Royal Children’s Hospital, Parkville, Australia; 4 Department of Paediatrics, University of Melbourne, Melbourne, Australia; 5 Radboud Center for Infectious Diseases, Radboud University Medical Center, P.O. Box 9101, 6500 HB Nijmegen, The Netherlands

**Keywords:** Q fever fatigue syndrome, Chronic fatigue syndrome, *MT*-*RNR1*, *MT*-*RNR2*, Humanin, MOTS-c

## Abstract

**Background:**

Q fever fatigue syndrome (QFS) is a well-documented state of prolonged fatigue following around 20% of acute Q fever infections. It has been hypothesized that low grade inflammation plays a role in its aetiology. In this study, we aimed to identify transcriptome profiles that could aid to better understand the pathophysiology of QFS.

**Methods:**

RNA of monocytes was collected from QFS patients (n = 10), chronic fatigue syndrome patients (CFS, n = 10), Q fever seropositive controls (n = 10), and healthy controls (n = 10) who were age- (± 5 years) and sex-matched. Transcriptome analysis was performed using RNA sequencing.

**Results:**

Mitochondrial-derived peptide (MDP)-coding genes *MT*-*RNR2* (humanin) and *MT*-*RNR1* (MOTS-c) were differentially expressed when comparing QFS (− 4.8 log2-fold-change *P* = 2.19 × 10^−9^ and − 4.9 log2-fold-change *P* = 4.69 × 10^−8^), CFS (− 5.2 log2-fold-change, *P* = 3.49 × 10^−11^ − 4.4 log2-fold-change, *P* = 2.71 × 10^−9^), and Q fever seropositive control (− 3.7 log2-fold-change *P* = 1.78 × 10^−6^ and − 3.2 log2-fold-change *P* = 1.12 × 10^−5^) groups with healthy controls, resulting in a decreased median production of humanin in QFS patients (371 pg/mL; Interquartile range, IQR, 325–384), CFS patients (364 pg/mL; IQR 316–387), and asymptomatic Q fever seropositive controls (354 pg/mL; 292–393).

**Conclusions:**

Expression of MDP-coding genes *MT*-*RNR1* (MOTS-c) and *MT*-*RNR2* (humanin) is decreased in CFS, QFS, and, to a lesser extent, in Q fever seropositive controls, resulting in a decreased production of humanin. These novel peptides might indeed be important in the pathophysiology of both QFS and CFS.

**Electronic supplementary material:**

The online version of this article (10.1186/s12967-019-1906-3) contains supplementary material, which is available to authorized users.

## Background

Q fever is a zoonotic disease caused by the intracellular Gram-negative bacterium *Coxiella burnetii*. Humans usually become infected through inhalation of infected aerosols that arise from small ruminants, of which parturient fluids are most notorious [1]. The bacterium primarily infects alveolar macrophages [1, 2]. By subverting host cell functions such as Toll-like receptor (TLR) recognition, apoptosis, and vesicular trafficking, *C. burnetii* is able to survive and replicate inside the phagolysosome of monocytes and macrophages [2]. Once inside the phagolysosome, the *Coxiella* Containing Vacuole (CCV), *C. burnetii* employs a Dot/Icm type IV secretion system through which it manipulates host cell processes [3, 4]. It is assumed that immune competent individuals are able to clear the infection eventually, making Q fever a self-limiting disease.

Initial infection with *C. burnetii* leads to symptomatic disease, i.e., acute Q fever, in around 40% of cases. This often presents as a flu-like illness, which is sometimes accompanied by pneumonia or hepatitis [2]. Of all those who become infected, around 1–5% develop a persistent infection with *C. burnetii*, chronic Q fever or persistent focalised infection, usually manifesting as endocarditis or infection of pre-existing aneurysms or vascular prostheses [1, 5]. Q fever fatigue syndrome (QFS) occurs in around 20% of symptomatic acute Q fever infections. QFS is characterised by a state of prolonged fatigue that lasts at least 6 months and often coincides with several other complaints, leading to substantial disabilities [6].

Complaints such as fatigue, musculoskeletal pain, headache, night sweating and recurrent upper respiratory tract infections suggest an inflammatory component in QFS. Active infection has however not been convincingly shown in these patients. In accordance with this, the first randomized placebo-controlled trial for QFS treatment was recently published, comparing cognitive behavioural therapy (CBT) and doxycycline with placebo treatment, demonstrating a beneficial effect for CBT, but not doxycycline, in reducing fatigue severity at end of treatment [7]. In 1998, Pentilla et al. published that peripheral blood mononuclear cells (PBMCs) of QFS patients produce significantly more interleukin (IL)-6 than cells of various control groups when exposed to Q fever antigens [8]. Our group has demonstrated that QFS patients show signs of altered immunity through monocyte-derived cytokines tumor necrosis factor (TNF)α, IL-1β, and especially IL-6, together with the interferon (IFN)γ- axis [9–11].

Chronic fatigue syndrome (CFS) is a disease with a striking overlap in symptoms with QFS that shows a subtle difference in psychological perpetuating factors and inflammatory profile [11, 12]. As in QFS, the aetiology of CFS remains unclear and is thought to involve the immune system [11, 13, 14], possibly through, or in combination with, mitochondrial dysfunction [15–17]. Complaints such as fatigue and exercise intolerance in CFS, but also QFS, strengthen the theory that mitochondrial dysfunction is involved in its pathogenesis.

To further elucidate the pathophysiology of chronic fatigue syndromes such as QFS and CFS, we used next-generation sequencing to investigated the transcriptomes of unstimulated circulating monocytes of QFS patients, CFS patients, asymptomatic Q fever seropositive controls, and healthy controls, all matched for age and sex.

## Methods

### Study population

The study population consisted of QFS patients (n = 10), CFS patients (n = 11), asymptomatic Q fever seropositive controls (n = 10), and healthy controls (n = 10), matched for age (± 5 years) and sex.

All QFS patients were diagnosed at the Radboud Expert Center for Q fever, Nijmegen, the Netherlands, after a uniform work-up according to the Dutch guideline on QFS [18]. All QFS patients met the following diagnostic criteria: (i) fatigue lasted ≥ 6 months; (ii) sudden onset of severe fatigue (defined as a score ≥ 35 on the subscale fatigue severity of the Checklist Individual Strength (CIS) questionnaire) [19] (Additional file 1: Table S1), or significant increase in fatigue, both related to a symptomatic acute Q fever infection; iii. chronic Q fever and other somatic or psychiatric causes of fatigue were excluded; and iv. fatigue resulted in significant functional impairment (defined as a total score ≥ 450 on the Sickness Impact Profile-8 (SIP-8) questionnaire) [20] (Additional file 1: Table S2).

All CFS patients were diagnosed at the Department of Internal Medicine and Expert Center for Chronic Fatigue (ECCF) of the Radboud university medical center, Nijmegen, the Netherlands, after a uniform work-up according to the Centers for Disease Control (CDC) criteria for CFS [21], strengthened with scores on SIP-8 and CIS, subscale on fatigue severity, questionnaires. All CFS patients tested negative on Q fever serology (Immunofluorescence assay, or IFA; Focus Diagnostics, Cypress, CA, USA) and, additionally, had a score ≥ 35 on the subscale fatigue severity of the CIS questionnaire and a score ≥ 450 on the SIP-8 questionnaire.

Asymptomatic Q fever seropositive controls were asked to participate by the primary investigator (RR); all of them tested positive on Q fever serology ≥ 5 years after the Q fever outbreak that took place between 2007 and 2011 (IgG phase I or II ≥ 1:16, but IgG phase I < 512), and reported no complaints of fatigue or functional impairment.

Colleagues from the Department of Internal Medicine at the Radboud university medical center, Nijmegen, who lived in areas previously endemic for Q fever during the Dutch outbreak between 2007 and 2011, tested negative on Q fever serology (IFA), and reported no complaints of fatigue or functional impairment, were asked to participate by the primary investigator (RR) as healthy controls.

### PBMC and monocyte isolation

PBMC were isolated by dilution of blood in PBS (1:1) and fractions were separated by density centrifugation over Ficoll-Paque (Ficoll-Paque Plus; GE healthcare, Zeist, The Netherlands). Cells were washed three times with cold PBS and resuspended in RPMI 1640 Dutch modification culture medium (Life Technologies/Invitrogen, Breda, The Netherlands) supplemented with 50 μg/mL gentamicin, 2 mM Glutamax, and 1 mM pyruvate (Life Technologies). Percoll isolation of monocytes was performed as previously described [22]. Briefly, 150–200 × 10^6^ PBMCs were layered on top of a hyper-osmotic Percoll solution (48.5% Percoll, 41.5% sterile H_2_O, 0.16 M filter sterilized NaCl) and centrifuged for 15 min at 580*g*. The interphase layer was isolated and cells were washed once with cold PBS. Cells were resuspended in culture medium as described above.

### PBMC stimulation and peptide assays

PBMCs were plated in 96-well round-bottom plates (Corning) at a concentration of 5 × 10^5^/mL in a total volume of 200 µL. The samples were stimulated with 10 ng/mL lipopolysaccharide (LPS) (Sigma-Aldrich, St. Louis, MO; from *E. coli*) for 24 h at 37 °C with 5% CO_2_. After stimulation, supernatants were collected and stored at − 20 °C until peptide assays were performed. Humanin and MOTS-c were measured using enzyme-linked immune sorbent assay (ELISA) according to the manufacturer protocol (MyBioSource, San Diego, CA, USA).

### RNA isolation and quantification

RNA was isolated from the monocyte-enriched suspension using the mirVana™ miRNA Isolation Kit (Ambion, Austin, TX, USA) according to the manufacturer’s instructions. The purity and quantity of RNA were assessed using NanoDrop software, after which samples were immediately stored at − 80 °C for future use.

### RNA sequencing alignment and expression analysis

RNA sequencing was performed on monocytes from all patients and transcriptome data is available at Gene Expression Omnibus (GSE130353) [23]. Reads were aligned using GSNAP [24], using non-default parameters −m 1 −N 1 −n 1 −Q −s Ensembl_splice_68. RNA sequencing library data were initially subjected to a quality control step, where, based on read distribution over the annotated genome, libraries that are outliers were identified and discarded from further analysis. For expression analyses reads were aligned to the Ensembl v68 human transcriptome using Bowtie. Quantification of gene expression was performed using MMSEQ.

### Differential expression

Analysis was performed using DESeq2, using as input the MMSEQ counts (Unique hits). Only genes with average counts above 1 were fed into the DESeq2 software. DESeq2 internally performs another round of independent filtering (usually the filter threshold of average number of reads would be ~ 5). To check for the main source of variation, principal component analysis (PCA) was performed on the top 500 varying genes (Additional file 1: Table S3).

### Pathway enrichment analysis

For the analysis of over- and underrepresented biological pathways, comparing the various groups (n = 10), the ClueGo V2.1.7 appliance was used in the integrative bio-informatic software environment of Cytoscape V3.2. Pathways were significantly overexpressed if they showed a *P* value ≤ 0.05 and only differentially expressed genes with a *P* value < 0.01 were selected for pathway analysis. Various analysis pathways like Kyoto Encyclopedia of Genes and Genomes pathway (KEGG), Reactome pathway, and Wikipathway were used on the differentially expressed gene lists, followed by Benjamini–Hochberg adjustment for terms and groups.

### Statistical analysis

Patient characteristics data were analyzed using Graphpad Prism (Graphpad Software Inc., version 5.03) and SPSS (Version 22.0, SPSS, Inc). The Mann–Whitney and Kruskal–Wallis test were used as non-parametric *t*-test and ANOVA to determine differences between groups. Statistical significance was attained if *P* < 0.05.

### Ethical statement

All participants provided written informed consent and the study was approved by the Medical Ethical Review Committee of the Arnhem-Nijmegen region.

## Results

### Patients and controls

At the time of blood collection, the median symptom duration of QFS and CFS did not differ significantly (*P* = 0.27), nor did the median age (*P* = 0.95) and gender of all groups (Table 1). All QFS patients and asymptomatic Q fever seropositive controls had IgG phase I or phase II titres ≥ 1:16, but IgG phase I ≤ 1:512, and none of them showed serological signs of an acute or recent Q-fever infection, reflected by IgM antibodies in absence of IgG antibodies.

**Table 1:** Characteristics of healthy controls, chronic fatigue syndrome (CFS) patients, Q fever fatigue syndrome (QFS) patients, and asymptomatic Q fever seropositive controls

Characteristics	Healthy controls (n = 10)	CFS (n = 10)	QFS (n = 10)	Q fever seropositives (n = 10)
Male sex, number (%)	5 (50)	5 (50)	5 (50)	5 (50)
Age, years Median (IQR)	54 (37–61)	48 (41–52)	43 (40–58)	50 (43–57)
Duration of symptoms, months^a^ Median (IQR)	–	110 (31–253)	78 (62–87)	–
CIS subscale fatigue severity score, mean ± SD	–	51 ± 5.1	52 ± 4.1	–
SIP-8 total score, mean ± SD	–	1600 ± 735.0	1474 ± 483.7	–

*QFS* Q fever fatigue syndrome, *CFS* chronic fatigue syndrome, *IQR* interquartile range; *CIS* Checklist Individual Strength, *SD* standard deviation, *SIP*-*8* Sickness Impact Profile-8

^a^Symptom duration: time onset of symptoms until blood sampling

### PCA and pathway enrichment analysis

**Fig. 1:**
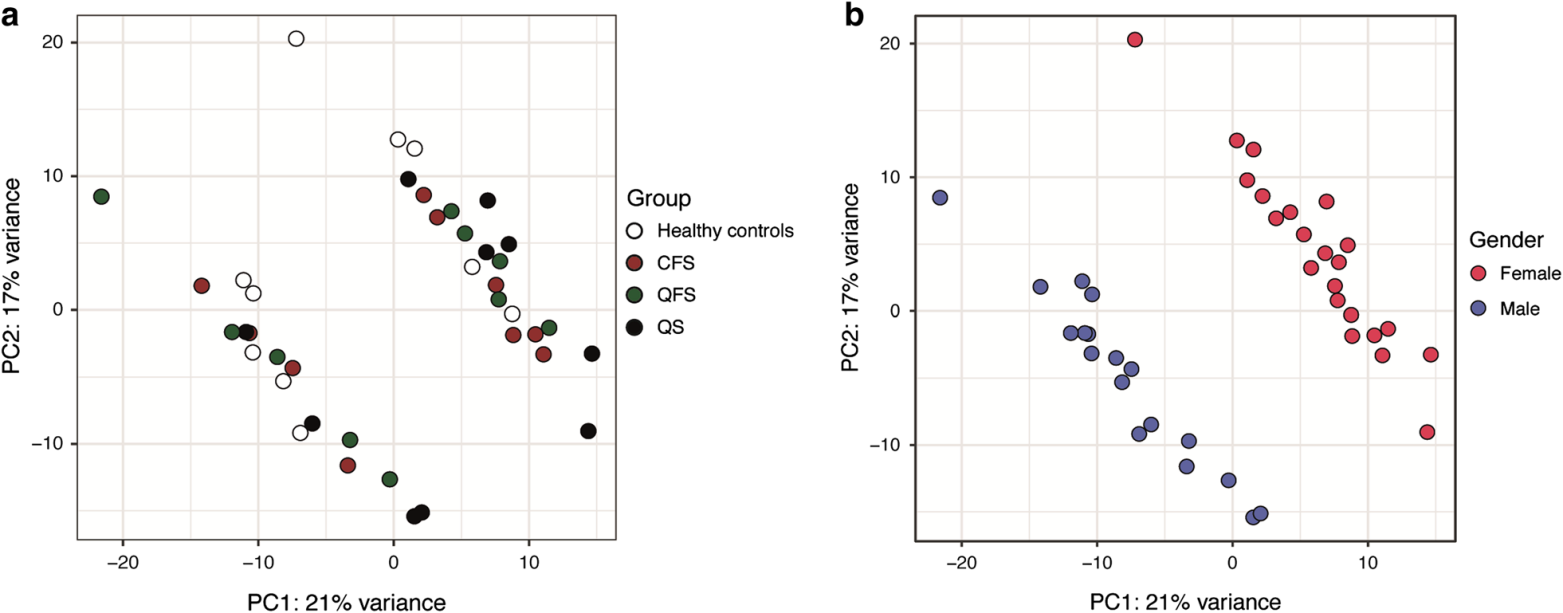
Principal-component analysis of top 500 varying genes. Principal component analysis of RNA sequencing data obtained from the top 500 varying genes in unstimulated, circulating monocytes of healthy controls (n = 10), CFS patients (n = 10), QFS patients (n = 10), and asymptomatic Q fever seropositive controls (n = 10), finding no variation between groups (**a**). The variation that is seen is based on sex (**b**). Principal Component (PC)1 (x-axis) represents 21% and PC2 (y-axis) represents 17% of total variation in data. *PC* principal component, *CFS* chronic fatigue syndrome, *QFS* Q fever fatigue syndrome, *QS* (*Q fever seropositives*), asymptomatic Q fever seropositive controls

A PCA was performed on the top 500 varying genes and found no difference between groups (Fig. 1a), except for sex [25] (Fig. 1b). Pathway enrichment analysis was performed on differentially expressed genes, using a cut-off *P* value ≤ 10 × 10^−5^, yielding no results. Pathway enrichment analysis was then repeated, using a cut-off *P* value ≤ 0.01. Although no apparent immunologic activation or inhibition was seen when comparing various groups, some mitochondrial alterations were observed (Additional file 1: Table S4A–E).

### Genes of interest

**Fig. 2:**
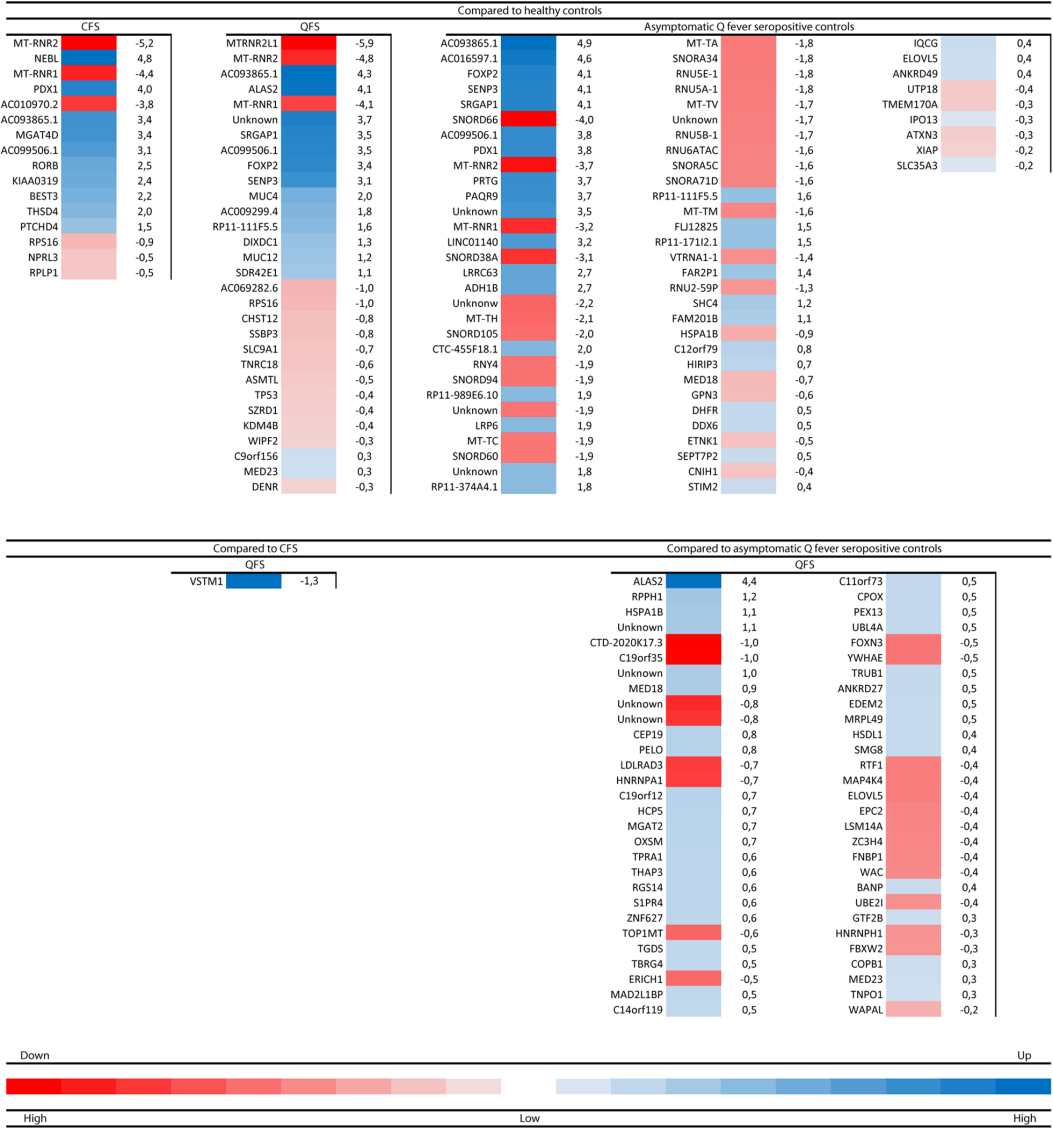
Differential gene expression in chronic fatigue syndrome (CFS) patients, Q fever fatigue syndrome (QFS) patients, and asymptomatic Q fever seropositive controls, compared to healthy controls, and QFS patients compared to CFS patients and Q fever seropositive controls. Heatmaps showing differential expression of down- and upregulated genes in circulating monocytes with a cut-off *P* value ≤ 10 × 10^−5^. Down- and upregulated genes in CFS patients (n = 10), QFS patients (n = 10), and asymptomatic Q fever seropositive controls (n = 10), compared to healthy controls (n = 10), down- and up-regulated genes in QFS patients (n = 10) compared to CFS patients (n = 10), and down- and upregulated genes in QFS patients (n = 10) compared to asymptomatic Q fever seropositive controls (n = 10). Heatmaps are based on level of differential expression, measured with log 2 fold change and depicted next to each gene, compared to various control groups. *CFS* chronic fatigue syndrome, *QFS* Q fever fatigue syndrome, *QS* asymptomatic Q fever seropositive controls

Using a cut-off differential expression value > and < 2-fold change, together with a cut-off *P* value ≤ 10 × 10^−5^, on both up- and down-regulated genes, several appear to be of interest (Fig. 2). When comparing CFS to healthy controls, those are *MT*-*RNR2* (− 5.2 log 2 fold change; *P* = 3.49 × 10^−11^)*, MT*-*RNR1* (− 4.4 log 2 fold change; *P* = 2.71 × 10^−9^) and *AC010970.2* (− 3.8 log 2 fold change; *P* = 1.28 × 10^−5^) for the down- regulated genes and *NEBL* (4.8 log 2 fold change; *P* = 2.79 × 10^−5^)*, PDX1* (3.9 log 2 fold change; *P* = 1.71 × 10^−5^)*, AC093865.1* (3.4 log 2 fold change; *P* = 1.07 × 10^−5^)*, MGAT4D* (3.4 log 2 fold change; *P* = 5.33 × 10^−5^)*, AC099506.1* (3.1 log 2 fold change; *P* = 1.52 × 10^−5^)*, RORB* (2.5 log 2 fold change; *P* = 2.93 × 10^−5^)*, KIAA0319* (2.4 log 2 fold change; *P* = 4.02 × 10^−6^), and *BEST3* (2.2 log 2 fold change; *P* = 9.93 × 10^−5^) for the up-regulated genes. When comparing QFS to healthy controls, those are *MT*-*RNR2* (− 4.8 log 2 fold change; *P* = 2.19 × 10^−9^)*, MT*-*RNR1* (− 4.9 log 2 fold change; *P* = 4.69 × 10^−8^), and MTRNR2L1 (− 5.9 log 2 fold change; *P* = 2.88 × 10^−5^) for the down-regulated genes and *AC093865.1* (4.8 log 2 fold change; *P* = 6.73 × 10^−8^)*, ALAS2* (4.1 log 2 fold change; *P* = 4.64 × 10^−5^)*, SRGAP1* (3.5 log 2 fold change; *P* = 8.09 × 10^−7^)*, AC099506.1* (3.5 log 2 fold change; *P* = 1.47 × 10^−6^)*, FOXP2* (3.4 log 2 fold change; *P* = 1.51 × 10^−5^)*, SENP3* (3.1 log 2 fold change; *P* = 9.69 × 10^−6^), and *MUC4* (2.0 log 2 fold change; *P* = 6.16 × 10^−7^) for the up-regulated genes. When comparing asymptomatic Q fever seropositive controls to healthy controls, those are *SNORD66* (− 4.0 log 2 fold change; *P* = 8.71 × 10^−7^)*, MT*-*RNR2* (− 3.7 log 2 fold change; *P* = 1.78 × 10^−6^)*, MT*-*RNR1* (− 3.2 log 2 fold change; *P* = 1.12 × 10^−5^)*, SNORD38A* (− 3.1 log 2 fold change; *P* = 4.51 × 10^−5^)*, MT*-*TH* (− 2.1 log 2 fold change; *P* = 1.92 × 10^−5^), and *SNORD105* (− 2.0 log 2 fold change; *P* = 6.94 × 10^−5^) for the down-regulated genes and *AC093865.1* (4.9 log 2 fold change; *P* = 2.19 × 10^−10^)*, AC016597.1* (4.6 log 2 fold change; *P* = 9.16 × 10^−7^)*, FOXP2* (4.2 log 2 fold change; *P* = 5.30 × 10^−8^)*, SENP3* (4.1 log 2 fold change; *P* = 4.1 × 10^−9^)*, SRGAP1* (4.1 log 2 fold change; *P* = 6.42 × 10^−9^)*, AC099506.1* (3.8 log 2 fold change; *P* = 5.69 × 10^−8^)*, PDX1* (3.8 log 2 fold change; *P* = 4.38 × 10^−5^)*, PRTG* (3.7 log 2 fold change; *P* = 6.04 × 10^−7^)*, PAQR9* (3.7 log 2 fold change; *P* = 9.17 × 10^−5^)*, LINC01140* (3.2 log 2 fold change; *P* = 2.46 × 10^−5^)*, LRRC63* (2.7 log 2 fold change; *P* = 2.34 × 10^−5^)*, ADH1B* (2.7 log 2 fold change; *P* = 9.47 × 10^−5^), and *CTC*-*455F18.1* (2.0 log 2 fold change; *P* = 9.38 × 10^−5^) for the up-regulated genes. When comparing QFS to asymptomatic Q fever seropositive controls, this is *ALAS2* (4.4 log 2 fold change; *P* = 1.81 × 10^−5^) for the up-regulated genes.

### Expression of *MT-RNR1 *and *MT-RNR2*

**Fig. 3:**
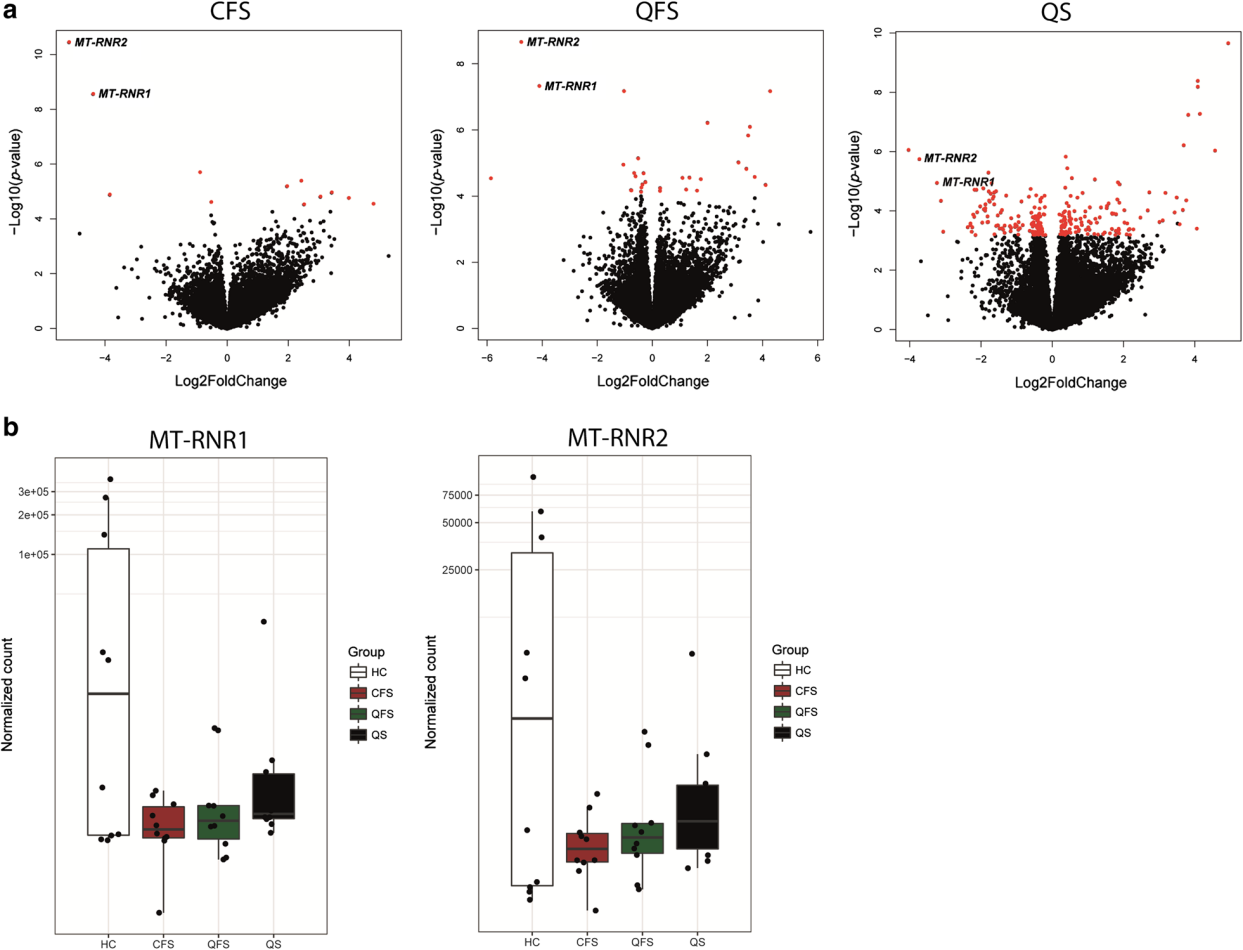
Volcano- and boxplots showing differential expression of *MT*-*RNR1* and *MT*-*RNR2* in chronic fatigue syndrome (CFS) patients, Q fever fatigue syndrome (QFS) patients, and asymptomatic Q fever seropositive controls, compared to healthy controls. Volcanoplots showing differential expression of *MT*-*RNR1* and *MT*-*RNR2* in circulating monocytes of CFS patients (n = 10), QFS patients (n = 10), and asymptomatic Q fever seropositive controls (n = 10), compared to healthy controls (n = 10) (**a**) and boxplots showing normalised counts of *MT*-*RNR1* and *MT*-*RNR2* in circulating monocytes of CFS patients (n = 10), QFS patients (n = 10), asymptomatic Q fever seropositive controls (n = 10), and healthy controls (n = 10) (**b**) *CFS* chronic fatigue syndrome, *QFS* Q fever fatigue syndrome, *QS* (*Q fever seropositives*) asymptomatic Q fever seropositive controls

Heatmaps comparing various groups were made depicting genes that are differentially expressed, using a cut-off *P* value ≤ 10 × 10^−5^ (Fig. 2). If we consider down regulated genes in patients compared to healthy controls, two genes, namely *MT*-*RNR1* and *MT*-*RNR2*, were consistently less expressed in CFS patients (− 4.4 log 2 fold change; *P* = 2.71 × 10^−9^, and − 5.2 log 2 fold change; *P* = 3.49 × 10^−11^, respectively), QFS patients (− 4.9 log 2 fold change; *P* = 4.69 × 10^−8^, and − 4.8 log 2 fold change; *P* = 2.19 × 10^−9^, respectively), and asymptomatic Q fever seropositive controls (− 3.2 log 2 fold change; *P* = 1.12 × 10^−5^, and − 3.7 log 2 fold change; *P* = 1.78 × 10^−6^, respectively) (Figs. 2, 3). Expression of *MT*-*RNR1* and *MT*-*RNR2* in individual patients and controls shows that there is a natural variation of expression in healthy controls, whilst patient groups all show low expression. The median expression is slightly higher in asymptomatic Q fever seropositive controls group compared to QFS and CFS (Fig. 3b).

### Production of MOTS-c and humanin

**Fig. 4:**
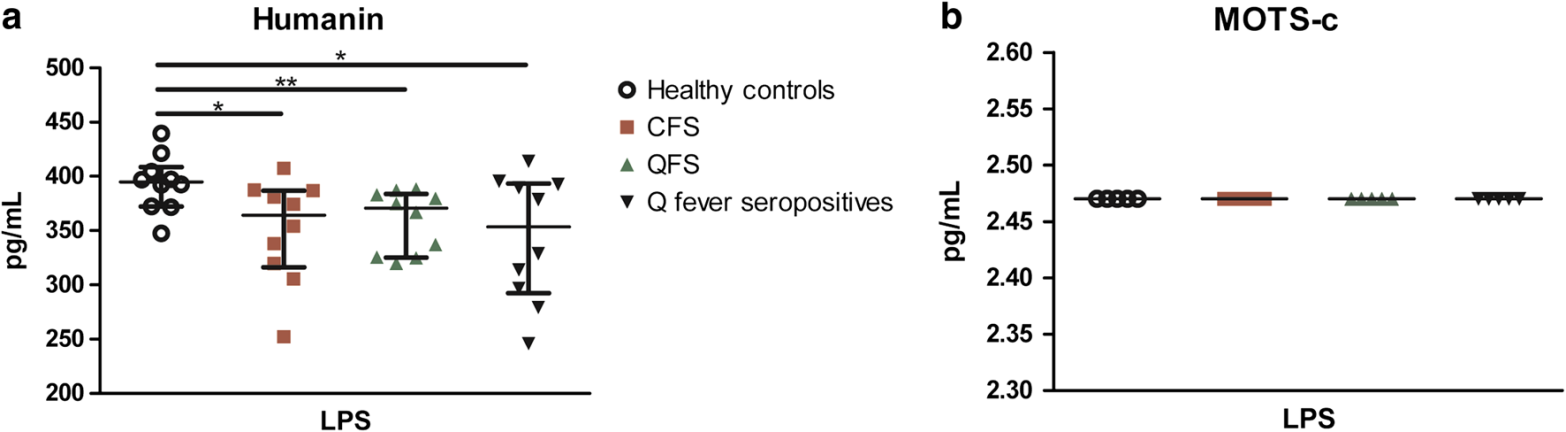
Production of mitochondrial-derived peptides (MDP) humanin and MOTS-c after 24 h stimulation of PBMCs with LPS. LPS-induced median MDP production after 24 h incubation of PBMCs of healthy controls (n = 10), CFS patients (n = 10), QFS patients (n = 10), and asymptomatic Q fever seropositive controls (n = 10). **a** LPS-induced median humanin production after 24 h incubation of PBMCs, showing a significant difference in humanin production between healthy controls (395 pg/mL; 372–409) and CFS patients (364 pg/mL; IQR 316–387), QFS patients (371 pg/mL; IQR 325–384), and asymptomatic Q fever seropositive controls (354 pg/mL; 292–393) (P = 0.05). **b** LPS-induced median MOTS-c production after 24 h incubation of PBMCs, showing no significant difference in MOTS-c production between healthy controls and CFS patients, QFS patients, and asymptomatic Q fever seropositive controls. Data are depicted as median with IQR. *QFS* Q fever fatigue syndrome, *CFS* chronic fatigue syndrome, *Q fever seropositives* Q fever seropositive controls, *LPS* lipopolysaccharide, *MDP* mitochondrial-derived peptide, *PBMC* peripheral blood mononuclear cell, *IQR* interquartile range. **P ≤ 0.01, *P ≤ 0.05

Mitochondrial-derived peptides (MDPs) MOTS-c and humanin, respectively encoded by *MT*-*RNR1* and *Mt*-*RNR2*, were measured after stimulation with LPS for 24 h. Stimulation of PBMCs with LPS resulted in a decreased median production of humanin in QFS patients (371 pg/mL; IQR 325–384), CFS patients (364 pg/mL; IQR 316–387), and asymptomatic Q fever seropositive controls (354 pg/mL; 292–393), compared to healthy controls (395 pg/mL; 372–409) (*P* = 0.05) (Fig. 4a). No difference between groups was found for MOTS-c production (Fig. 4b).

## Discussion

When looking at disease, we found that the transcriptomes of circulating monocytes in QFS patients are not grossly different from those of healthy controls, asymptomatic Q fever seropositive controls, and CFS patients. Among the limited differences observed, MDP-coding genes *MT*-*RNR1* (MOTS-c) and *MT*-*RNR2* (humanin) [26, 27] were significantly less expressed (*P* < 1 × 10^−5^) in all groups of patients as compared to control subjects. To further substantiate this finding we stimulated PBMCs of these patients with LPS for 24 h and found that this resulted in lower production of humanin, but not MOTS-c, in CFS patients, QFS patients, and asymptomatic Q fever seropositive controls, compared to healthy controls.

Humanin and MOTS-c are MDPs and as such may act through retrograde signalling, but can also serve in an intracellular and endocrine manner [28]. Humanin is the first ever described MDP and, studies have shown that it mainly acts in an anti-apoptotic and cytoprotective manner. It has also been implicated in the regulation of metabolism and inflammation [29]. Humanin binds to the receptor complex CNTFR/WSX-1/gp130, of which gp130 is a common subunit of receptors belonging to the IL-6 receptor family [30]. Subsequent activation of JAK/STAT, AKT and ERK pathways are thought to play a role in its cytoprotective effects [26, 30]. Zao et al. have shown that LPS stimulation of astrocytes isolated from rats, pre-incubated with humanin for 24 h resulted in a decreased production of IL-6, TNFα, and IL-1β [31]. In our hands, previous stimulation experiments of PBMCs with LPS for 24 h did not result in a difference in IL-6, TNFα, or IL-1β production between CFS patients, QFS patients, asymptomatic Q fever seropositive controls, and healthy controls. However, this does not to mean that role of humanin in IL-6, TNFα, and IL-1β production has to be discarded. Zao et al. pre-incubated astrocytes with extracellular humanin for 24 h before stimulating with LPS. This is an experimental setting completely different from ours. As we did not find a difference in concentrations of circulating humanin between CFS patients, QFS patients, asymptomatic Q fever seropositive controls, and healthy controls (data not shown), it is unlikely that isolated PBMCs were exposed to different concentrations of humanin before LPS stimulation. Furthermore, we do not have any insight in the kinetics of humanin production by stimulated PBMCs and monocytes. Further research on the kinetics and effects of humanin release by PBMCs, and especially monocytes, on cytokine production is needed.

MOTS-c is the second MDP that was discovered and has since been implicated in the regulation of insulin resistance and metabolic homeostasis, mainly through activation of AMPK [27, 29]. Despite the down-regulated transcription for this MDP, we did not find a difference in MOTS-c production between groups. In fact, we found very low extracellular production (2.47 pg/mL), which is the lower limit of the detection range. Although no previous studies have measured MOTS-c production following LPS stimulation, high levels of circulating MOTS-c have been found in blood plasma [32, 33], indicating constitutive extracellular production. It could be argued that MOTS-c production is regulated through mechanisms different to those tested in our experimental setup.

As was previously shown, PBMCs of QFS patients produce significantly more IL-6 than healthy controls, CFS patients, and asymptomatic Q fever seropositive controls, when stimulated with Q fever antigen [8]. This was also the case for IL-1β when comparing QFS patients with CFS patients and asymptomatic Q fever seropositive controls, and TNFα when comparing QFS patients with CFS patients and healthy controls. Interestingly, circulating IL-6 was also significantly increased in QFS patients compared to healthy controls, an observation that was made before in CFS patients [13, 34]. Looking at the transcriptomes of circulating monocytes in QFS and CFS patients, compared to healthy controls, no genes associated with the IL-6 production pathway seem to be overly expressed.

Not much is known on the exact mechanisms through which humanin and MOTS-c exert their effects in various diseases. Looking at known mechanisms, it could be argued that these peptides are important in the pathophysiology of both CFS and QFS [26, 27, 29]. Their role in cell metabolism supports the hypothesis that chronic fatigue might result from a hypometabolic state [35–37]. It has long been thought that mitochondrial pathology underlies chronic fatigue aetiology, as one of the key features of mitochondrial disease is severe fatigue [36, 38–41]. Recent studies showed that PBMCs of CFS patients show signs of impaired mitochondrial functioning compared to PBMCs of healthy controls when stressed [15, 16]. It was also found that CFS patients show mitochondrial genetic differences compared to healthy controls [17]. It would therefore be of great interest to further investigate the role of *MT*-*RNR1* and *MT*-*RNR2* in these discrepancies and mitochondrial dysfunction in chronic fatigue as a whole. Other than its role in regulating metabolism, humanin also serves as a neuroprotective factor that could potentially influence neuroinflammation by downplaying activation of microglia. This would fit with the PET-CT study by Nakatomy et al. in which CFS patients show signs of neuroinflammation [42]. A decreased expression of humanin might very well lead to the neuroinflammatory processes that were seen in these patients. These processes might explain the neurocognitive problems, e.g., memory loss, impaired concentration, etc., these patients often concomitantly experience [6]. Levels of circulating humanin and MOTS-c have been described to decline with age, an interesting observation as chronically fatigued patients often describe themselves as suddenly having aged significantly [27, 43]. MOTS-c is also known to regulate muscle metabolism and has been implicated in the regulation of exercise [27]. A deficiency of this peptide might therefore be involved in common complaints of muscle ache and exercise intolerance in CFS and QFS.

It is intriguing that *MT*-*RNR1* and *MT*-*RNR2* were less expressed in asymptomatic Q fever seropositive controls, albeit to a lesser extent than in QFS and CFS patients. It is conceivable that an acute Q fever infection leads to a decreased expression of these genes, Raijmakers, 2018 (unpublished data), which is then maintained in the long term, possibly through epigenetic remodeling [44]. The fact that some acute Q fever patients remain fatigued following their infection could suggest that a concomitant, perhaps psychological, incentive is needed to induce a clinical chronic fatigue syndrome.

In addition to the differential expression of *MT*-*RNR1* and *MT*-*RNR2*, up-regulated genes *AC093865.1* and *AC099506.1* are of particular interest as they show potential to differentiate chronically fatigued patients as a whole from healthy controls. Another gene of interest is *ALAS2*, which is up-regulated in QFS patients compared to both healthy controls and asymptomatic Q fever seropositive controls, making it potentially specific for QFS. Expression of *ALAS2* regulates haem systhesis and is upregulated by haem and iron [45]. Loss-of-function mutations in this gene are associated with sideroblastic anaemias, while gain-of-function mutations are associated with porphyria [46, 47]. Interestingly, QFS patients often exhibit increased levels of ferritin [12]. It would be worthwhile to further investigate the role of *ALAS2* in the pathogenesis of QFS. A particularly crucial question would be whether up-regulation of this gene results in increased levels of ferritin or vice versa.

Other than the differential expression of *MT*-*RNR1* and *MT*-*RNR2*, no clear alterations were detected in the transcriptomes of circulating monocytes when comparing between various groups. This was however a scenario we accounted for as previous studies on chronic fatigue have only found subtle immunologic differences, which are then often contradicted by subsequent studies [13]. If there is indeed an immunologic component at play, it is likely to a subtle one. As previous stimulation experiments did result in differences in cytokine production and patients often experience setbacks following incentives such as infections and excessive exercise, it might be worthwhile to investigate the transcriptomes of stimulated, instead of circulating, monocytes.

## Conclusion

QFS patients, CFS patients, and, to a lesser extent, asymptomatic Q fever seropositive controls showed a decreased expression of MDP-coding genes *MT*-*RNR1* and *MT*-*RNR2*, resulting in a decreased production of humanin (*MT*-*RNR2*), compared to healthy controls. Whether these MDP-coding genes play a role in chronic fatigue syndromes such as CFS and QFS remains unclear. The fact that we find a decreased expression of these genes in asymptomatic Q fever seropositive controls as well is interesting. It could mean that these findings simply are not specific for chronic fatigue, or it could tell us that acute Q fever is not the only incentive needed to induce the clinical syndrome of chronic fatigue. Given the functions of MOTS-c and humanin, it is definitely worthwhile to further investigate the role of these MDP-coding genes in chronic fatigue.

## Additional file

**Additional file 1.** Additional tables.

## Data Availability

The datasets used and/or analysed during the current study are available from the corresponding author on reasonable request.

## Abbreviations

QFS: Q fever fatigue syndrome
CFS: chronic fatigue syndrome
MDP: mitochondrial-derived peptide
IQR: interquartile range
TLR: toll-like receptor
*C. burnetii*: *Coxiella burnetii*
CCV: *Coxiella* Containing Vacuole
CBT: cognitive behavioural therapy
PBMCs: peripheral blood mononuclear cells
IL: interleukin
IFN: interferon
TNF: tumor necrosis factor
CIS: Checklist Individual Strength (questionnaire)
SIP-8: Sickness Impact Profile-8 (questionnaire)
ECCF: Expert Center for Chronic Fatigue
CDC: Centers for Disease Control
IFA: immunofluorescence
LPS: lipopolysaccharide
PCA: principal component analysis
TNF: tumor necrosis factor
SD: standard deviation
