# Supplementary material for: A possible role for mitochondrial-derived peptides humanin and MOTS-c in patients with Q fever fatigue syndrome and chronic fatigue syndrome
Source: J Transl Med. 2019 May 14;17:157. doi: 10.1186/s12967-019-1906-3 (PMC6518812; doi:10.1186/s12967-019-1906-3)
Supplement: Supplementary file 1 — Additional file 1. Additional tables. [file 12967_2019_1906_MOESM1_ESM.doc]

**Table S1. Sickness Impact Profile (SIP)-8 questionnaire.**

| **Subscales** | **Range** | **Functionally impaired** |
| --- | --- | --- |
| **Sleep** | 0 - 499 | **Total score ≥ 450** |
| **Home management** | 0 - 668 |
| **Mobility** | 0 - 719 |
| **Social interaction** | 0 - 1450 |
| **Walking** | 0 - 842 |
| **Alertness** | 0 - 777 |
| **Work** | 0 - 1144 |
| **Pastime** | 0 - 422 |
| **Total score** | **0 - 6521** |

Patients are considered functionally impaired if Total score ≥ 450. Abbreviations: *SIP-8* = Sickness Impact Profile-8.

**Table S2. Checklist Individual Strength (CIS) questionnaire.**

| **Subscales** | **Range** | **Severely fatigued** |
| --- | --- | --- |
| **Fatigue** | **8 - 56** | **Fatigue ≥ 35** |
| **Concentration** | 5 - 35 |
| **Motivation** | 4 - 28 |
| **Physical activity** | 3 - 21 |
| **Total score** | 20 - 140 |

Patients are considered severely fatigued if subscale Fatigue ≥ 35. Abbreviations: *CIS* = Checklist Individual Strength.

**Table S3. List of top varying genes.**

| **Top 500 varying genes** | | | | | | | | | | | | | | | | | | | | | | | | | | | |
| --- | --- | --- | --- | --- | --- | --- | --- | --- | --- | --- | --- | --- | --- | --- | --- | --- | --- | --- | --- | --- | --- | --- | --- | --- | --- | --- | --- |
| XIST |  | | SCARNA7 |  | | IGLV1-51 |  | | TPT1P5 |  | | SNORA5A | | SNORA37 | |  | | MKI67 |  | | CEBPA |  | | NAMPT |  | | DACT1 |
| DDX3Y |  | | RPS28 |  | | GLDC |  | | FADS2 |  | | EEF1A1P5 |  | | PF4 |  | | IFI6 |  | | MX1 |  | | SNORA71D | | IL2RB | |
| RPS4Y1 |  | | IGHA1 |  | | IGHV3-7 |  | | SNORD116-18 | | IGKV1-16 | |  | | RNY3 |  | | LINC02503 | | SNORA11 | |  | | ISG15 |  | | SECTM1 |
| TXLNGY |  | | MSLN |  | | TMTC1 |  | | IGHG4 |  | | SNORD116-23 | | SEMA6B | |  | | CD79A |  | | EPHA1-AS1 | | ARL10 | |  | | GP9 |
| KDM5D |  | | IGLC2 |  | | ERAP2 |  | | SNORA50A | | IGLV8-61 | |  | | SLC4A10 |  | | SPATA2 |  | | SNORA79B | | SCARNA13 | | | NT5E | |
| UTY |  | | SNORA65 |  | | CLEC4F |  | | ABCA13 |  | | SNORD105B | | ZFP36 | |  | | COL4A3 |  | | ADGRE4P |  | | CFH |  | | NFKBIA |
| USP9Y |  | | RNA5-8SP6 | | IFIT1B | |  | | SNORD116-24 | | MDGA1 | |  | | AKAP12 |  | | MAB21L3 |  | | CD8B |  | | CXCL5 |  | | MT-ND4 |
| ZFY |  | | DEFA3 |  | | IGLV3-1 |  | | Z97192.2 |  | | MEG3 |  | | COX8A |  | | CCR7 |  | | NR4A1 |  | | CDKN1A |  | | CAVIN2 |
| AC010970.1 | | RNU4-1 | |  | | SNORD116-16 | | MT-TC | |  | | RGS1 |  | | SNORA66 |  | | JUN |  | | CHST13 |  | | AC127502.1 | | IGLV2-11 | |
| MT-RNR2 |  | | IGHG2 |  | | ALPL |  | | LINC02432 | | IFIT1 | |  | | ITGA2B |  | | SNORD14E | | SNORD9 | |  | | SYCP2L |  | | AC010468.1 |
| EIF1AY |  | | CA1 |  | | FCGR3B |  | | RNU2-3P |  | | LRRC37A4P | | AC025884.1 | | | AC135983.3 | | | MS4A2 | |  | | USP53 |  | | SCARNA1 |
| PRKY |  | | NAMPTP1 | | HLA-DQA1 | | | SNORD104 | | | PPBP | |  | | SNORD8 |  | | AC124312.3 | | SPARC | |  | | TOP2A |  | | ZNF860 |
| MT-RNR1 |  | | HBEGF |  | | PTGS2 |  | | FAM118A |  | | RNF150 |  | | MIR22HG |  | | BUB1 |  | | SPINK9 |  | | FGFBP2 |  | | NEAT1 |
| SENP3 |  | | HBA2 |  | | MTCO1P12 | | KRT72 | |  | | FCRL5 |  | | SNORD15A | | SNORA77 | |  | | CAMK4 |  | | MFSD10 |  | | MT-ND1 |
| HBB |  | | MMP8 |  | | RNU5B-1 |  | | HLA-J |  | | IGKV3-11 |  | | CD8A |  | | TXNDC5 |  | | DDIT4 |  | | ADGRG1 |  | | ANKRD36BP2 |
| NEBL |  | | ID1 |  | | IGHA2 |  | | FOS |  | | SLCO1A2 |  | | SNORA74A | | MALAT1 | |  | | SCARNA12 | | LINC02273 | | | AC010889.1 | |
| HLA-DQA2 | | CAMP | |  | | S100B |  | | AC020656.1 | | SERPINB10 | | | HLA-H | |  | | EREG |  | | SNORA80E | | HIGD2A | |  | | RNU1-11P |
| MAEL |  | | IGLC1 |  | | CCDC144A | | RF00012 | |  | | SIGLEC1 |  | | SNORA47 |  | | CHI3L1 |  | | ATOH8 |  | | TTYH3 |  | | AL671277.1 |
| MTRNR2L1 | | SNORD3B-1 | | | SNORD94 | |  | | VTRNA1-1 | | VSTM1 | |  | | SNORA49 |  | | TCN1 |  | | TENT5C |  | | MT-ATP6 |  | | TUBB1 |
| SRGAP1 |  | | RNU4-2 |  | | IFI44L |  | | MT-TV |  | | FCGR2C |  | | IGKV3-20 |  | | TACC2 |  | | RN7SL396P | | ARSA | |  | | SNORA73B |
| HLA-DQB1 | | RN7SKP180 | | | RNU2-61P | | | RN7SL151P | | | MT-TM | |  | | SOCS3 |  | | CYP27A1 |  | | L1TD1 |  | | IFITM3 |  | | CST3 |
| BCORP1 |  | | HLA-DRB6 | | HLA-G | |  | | AC007333.2 | | ITGB3 | |  | | MT-ATP8 |  | | GRN |  | | VNN1 |  | | IGHV4-39 |  | | SNORD90 |
| ALAS2 |  | | GPR15 |  | | IGKC |  | | LINC01609 | | IGLV1-44 | |  | | IL1B |  | | MAFF |  | | CEBPD |  | | IGKV4-1 |  | | TIMM17B |
| RNA5S9 |  | | LRRC63 |  | | ANXA3 |  | | REXO1L1P | | AC020916.1 | | | IGKV1-5 | |  | | CTSG |  | | GZMM |  | | PROK2 |  | | SPATA20 |
| RPS28P7 |  | | HBA1 |  | | IGLV2-14 |  | | SIGLEC14 |  | | NAPSB |  | | ATF3 |  | | GSTM3 |  | | SNORD89 |  | | LINC00989 | | CXCR1 | |
| TMEM176B | | RNU2-68P | | | H1F0 | |  | | RNU6ATAC | | MARCO | |  | | SNORA74B | | IER2 | |  | | IL3RA |  | | LDHAP7 |  | | CD40LG |
| RPL13P12 |  | | IGLV3-21 |  | | MT-TH |  | | DEFA4 |  | | SNORA2C |  | | SNORA2A | | AL139020.1 | | | LINC00599 | | | CES1 | |  | |  |
| G0S2 |  | | IGLC3 |  | | THBS1 |  | | SNORD38A | | AC129492.1 | | | AP001189.1 | | | ADM | |  | | LILRA4 |  | | SNORA75 |  | |  |
| LTF |  | | ESRG |  | | IGHM |  | | CALHM6 |  | | SNORA74D | | GNG11 | |  | | SNORD3A | | HP | |  | | IGLV3-9 |  | |  |
| EGR1 |  | | RPSAP15 |  | | AC024293.1 | | DUSP1 | |  | | RNU2-59P | | SNORA26 | |  | | LTBP1 |  | | CCDC144B | | GRINA | |  | |  |
| HLA-DRB5 | | SNORD66 | |  | | TSPO |  | | SCARNA10 | | COL18A1 | |  | | CSRNP1 |  | | SNORD15B | | HSPA7 | |  | | AL136987.1 | |  | |
| PRTG |  | | GSTM1 |  | | AC007952.4 | | SNORA38 | |  | | JUNB |  | | HDC |  | | ADH1B |  | | BPI |  | | EIF5B |  | |  |
| RPS2P46 |  | | CRISP3 |  | | RN7SKP80 | | DUSP2 | |  | | MMP25 |  | | NOG |  | | SNORA3B |  | | MYADM |  | | JAKMIP1 |  | |  |
| SNORD17 |  | | MT-TE |  | | SNORD116-15 | | DSP | |  | | COL5A2 |  | | MZB1 |  | | AC009299.1 | | SNORD100 | | | RPS16 | |  | |  |
| IGLV1-41 |  | | FOLR3 |  | | SNORA54 |  | | SNORA2B |  | | MME |  | | KLRC2 |  | | IFNG-AS1 |  | | LY6E |  | | MT-CO2 |  | |  |
| MYOM2 |  | | SNORA13 |  | | CEACAM8 | | OSM | |  | | APOBEC3B | | UGT8 | |  | | FAM153A |  | | SNORD99 |  | | MSR1 |  | |  |
| CXCL8 |  | | PLEKHS1 |  | | JUP |  | | AC020898.1 | | LINC02446 | | | SNORA46 | |  | | CHRM3-AS2 | | RPL9 | |  | | SH3BGRL2 | |  | |
| RPL21P28 |  | | RNU5A-1 |  | | SNORD60 |  | | NKG7 |  | | CEACAM6 | | C1QA | |  | | SNORA5C |  | | MMP9 |  | | RPL21P75 |  | |  |
| IGHG3 |  | | AC024940.2 | | HLA-DQB1-AS1 | | | LINC02520 | | | GATA2 | |  | | SCARNA3 |  | | MT-ND6 |  | | IGLV3-19 |  | | LYPD2 |  | |  |
| LINC00278 | | PAX8-AS1 | | | RNU2-64P | | | GPX1P1 | |  | | IGHGP |  | | TNNT1 |  | | SNORD117 | | RNU2-6P | |  | | SNORD12 |  | |  |
| IGHD |  | | IGLV2-23 |  | | TACSTD2 |  | | SNORD83A | | LCN2 | |  | | CUTALP |  | | CHI3L2 |  | | SNORA38B | | LRP3 | |  | |  |
| RNY1 |  | | JCHAIN |  | | AHRR |  | | SLC9A4 |  | | KRT73 |  | | SCARNA6 |  | | LINC01291 | | SNORA55 | |  | | LEF1 |  | |  |
| TMEM176A | | IGLV6-57 | |  | | SNORD69 |  | | SCARNA11 | | ANPEP | |  | | NLRP2 |  | | SNORA69 |  | | SNORD71 |  | | HMGB1P6 | |  | |
| OLFM4 |  | | RNU5E-1 |  | | SNORD46 |  | | GZMH |  | | NR4A2 |  | | MS4A3 |  | | ADTRP |  | | AL355075.4 | | WDR66 | |  | |  |
| FOXP2 |  | | IGHG1 |  | | TNFRSF17 | | SNORA33 | |  | | IGLV1-47 |  | | CLC |  | | KLF4 |  | | CPA3 |  | | C15orf54 |  | |  |
| SLC4A1 |  | | LRRN3 |  | | FOSB |  | | COL5A3 |  | | IGHV3-23 |  | | IGHV3-48 |  | | ARG1 |  | | STAB1 |  | | PLK3 |  | |  |
| RNY4 |  | | TMSB4Y |  | | SNORD3B-2 | | LGALS2 | |  | | TRGC2 |  | | SNORA20 |  | | RSAD2 |  | | SRRM2 |  | | LINC02397 | |  | |
| MT-TS2 |  | | HBD |  | | LRP6 |  | | IGLL5 |  | | TREML4 |  | | DDX11 |  | | DLGAP5 |  | | SIK1 |  | | PAX8 |  | |  |
| RN7SKP255 | | RNU5D-1 | |  | | ZFP57 |  | | RNU2-48P | | LOXHD1 | |  | | IFIT3 |  | | NPIPB15 |  | | IFI44 |  | | SCARNA5 |  | |  |

**Table S4. Pathway enrichment analysis of chronic fatigue syndrome (CFS) patients, Q fever fatigue syndrome (QFS) patients, and asymptomatic Q fever seropositive controls, compared to healthy controls, QFS patients compared to CFS patients, and QFS patients compared to asymptomatic Q fever seropositive controls.**

A. CFS compared to healthy controls, upregulated genes.

| Pathway | *P*-value | Enrichment |
| --- | --- | --- |
| GCNTs transfer GlcNAc from UDP-GlcNAc to Core 1 mucins | 0,00 | 2,74 |
| ST3GAL1-4 can add a sialic acid to the T antigen at the alpha 3 position | 0,00 | 2,71 |
| Diseases associated with O-glycosylation of proteins | 0,00 | 2,71 |
| Addition of galactose to Core 6 glycoprotein | 0,00 | 2,64 |
| C1GALT1 transfers Galactose to the Tn antigen forming Core 1 glycoproteins (T antigens) | 0,00 | 2,64 |
| ST6GALNAC3/4 can add a sialic acid to the sialyl T antigen to form the disialyl T antigen | 0,00 | 2,64 |
| Defective GALNT3 causes familial hyperphosphatemic tumoral calcinosis (HFTC) | 0,00 | 2,60 |
| Defective C1GALT1C1 causes Tn polyagglutination syndrome (TNPS) | 0,00 | 2,60 |
| Defective GALNT12 causes colorectal cancer 1 (CRCS1) | 0,00 | 2,60 |
| Defective GALNT12 does not transfer GalNAc to mucins | 0,00 | 2,60 |
| Defective GALNT3 does not transfer GalNAc to mucins | 0,00 | 2,60 |
| Defective C1GALT1C1 does not bind C1GALT1 | 0,00 | 2,60 |
| CLEC10A binds Tn-MUC1 | 0,00 | 2,60 |
| Sialyltransferase I can add sialic acid to the Tn antigen at the alpha 6 position | 0,00 | 2,60 |
| Sialyltransferase I can add sialic acid to the T antigen at the alpha 6 position | 0,00 | 2,60 |
| GalNAc alpha-2,6-sialyltransferase II can add a sialic acid to the T antigen at the alpha 6 position | 0,00 | 2,60 |
| Receptor-type tyrosine-protein phosphatases | 0,00 | 2,50 |
| Addition of GlcNAc to the Tn antigen forms a Core 3 glycoprotein | 0,00 | 2,50 |
| Termination of O-glycan biosynthesis | 0,00 | 2,47 |
| A4GNT transfers GlcNAc to core 2 mucins | 0,00 | 2,45 |
| CHST4 transfers SO4(2-) from PAPS to Core 2 mucins | 0,00 | 2,45 |
| Addition of GlcNAc to the Tn antigen via an alpha-1,3 linkage forms a Core 5 glycoprotein | 0,00 | 2,45 |
| Addition of galactose to the Tn antigen via an alpha-1,3 linkage forms a Core 8 glycoprotein | 0,00 | 2,45 |
| Addition of GlcNAc to the Tn antigen via a beta-1,6 linkage forms a Core 6 glycoprotein | 0,00 | 2,45 |
| Addition of GalNAc to the Tn antigen via an alpha-1,6 linkage forms a Core 7 glycoprotein | 0,00 | 2,45 |
| Addition of GlcNAc to Core 3 forms a Core 4 glycoprotein | 0,00 | 2,45 |
| Nicotine addiction | 0,00 | 2,32 |
| GALNTs transfer GalNAc from UDP-GalNAc to mucins to form Tn antigens | 0,00 | 2,32 |
| TRP channels | 0,00 | 2,31 |
| Dectin-2 family | 0,00 | 2,31 |
| Diseases of glycosylation | 0,00 | 2,31 |
| Axon guidance | 0,01 | 2,30 |
| TRPs transport extracellular Ca2+ to cytosol | 0,01 | 2,29 |
| O-linked glycosylation | 0,01 | 2,24 |

B. QFS compared to healthy controls, downregulated genes.

| Pathway | *P*-value | Enrichment |
| --- | --- | --- |
| RAC1:GTP:FMNL1 binds profilin:G-actin | 0,01 | 2,29 |
| SRGAP2 binds RAC1:GTP:FMNL1:profilin:G-actin | 0,01 | 2,29 |
| SRGAP2 stimulates RAC1 GTP-ase activity and ends FMNL1-mediated elongation of actin filaments | 0,01 | 2,29 |
| Host Interactions of HIV factors | 0,01 | 2,16 |
| Translocation of GLUT4 to the plasma membrane | 0,01 | 2,13 |
| p-AMPK:AMP phosphorylates Raptor in the mTORC1 complex | 0,01 | 2,12 |
| Platelet activation, signaling and aggregation | 0,01 | 2,11 |
| Platelet activation, signaling and aggregation | 0,01 | 2,11 |
| Platelet activation, signaling and aggregation | 0,01 | 2,11 |
| TP53 Regulates Metabolic Genes | 0,01 | 2,10 |
| Adherens junction | 0,01 | 2,07 |
| Pancreatic secretion | 0,01 | 2,05 |
| Viral carcinogenesis | 0,01 | 2,03 |
| RHO GTPase Effectors | 0,01 | 2,01 |
| Synthesis of PIPs at the Golgi membrane | 0,01 | 2,01 |
| Regulation of actin dynamics for phagocytic cup formation | 0,01 | 2,01 |
| RHO GTPases activate PKNs | 0,01 | 2,01 |
| Regulation of actin dynamics for phagocytic cup formation | 0,01 | 2,01 |
| RHO GTPases activate PKNs | 0,01 | 2,01 |

QFS compared to healthy controls, upregulated genes.

| Pathway | *P*-value | Enrichment |
| --- | --- | --- |
| KRAB-ZNF / KAP Interaction | 0,00 | 7,29 |
| Erythrocytes take up oxygen and release carbon dioxide | 0,00 | 6,06 |
| A4GNT transfers GlcNAc to core 2 mucins | 0,00 | 5,32 |
| CHST4 transfers SO4(2-) from PAPS to Core 2 mucins | 0,00 | 5,32 |
| Addition of GlcNAc to the Tn antigen via an alpha-1,3 linkage forms a Core 5 glycoprotein | 0,00 | 5,32 |
| Addition of galactose to the Tn antigen via an alpha-1,3 linkage forms a Core 8 glycoprotein | 0,00 | 5,32 |
| Addition of GlcNAc to the Tn antigen via a beta-1,6 linkage forms a Core 6 glycoprotein | 0,00 | 5,32 |
| Addition of GalNAc to the Tn antigen via an alpha-1,6 linkage forms a Core 7 glycoprotein | 0,00 | 5,32 |
| Addition of GlcNAc to Core 3 forms a Core 4 glycoprotein | 0,00 | 5,32 |
| Erythrocytes take up carbon dioxide and release oxygen | 0,00 | 5,22 |
| O2/CO2 exchange in erythrocytes | 0,00 | 5,22 |
| Defective GALNT3 causes familial hyperphosphatemic tumoral calcinosis (HFTC) | 0,00 | 5,22 |
| Defective C1GALT1C1 causes Tn polyagglutination syndrome (TNPS) | 0,00 | 5,22 |
| Defective GALNT12 causes colorectal cancer 1 (CRCS1) | 0,00 | 5,22 |
| Defective GALNT12 does not transfer GalNAc to mucins | 0,00 | 5,22 |
| Defective GALNT3 does not transfer GalNAc to mucins | 0,00 | 5,22 |
| Defective C1GALT1C1 does not bind C1GALT1 | 0,00 | 5,22 |
| CLEC10A binds Tn-MUC1 | 0,00 | 5,22 |
| Sialyltransferase I can add sialic acid to the Tn antigen at the alpha 6 position | 0,00 | 5,22 |
| Sialyltransferase I can add sialic acid to the T antigen at the alpha 6 position | 0,00 | 5,22 |
| GalNAc alpha-2,6-sialyltransferase II can add a sialic acid to the T antigen at the alpha 6 position | 0,00 | 5,22 |
| Addition of galactose to Core 6 glycoprotein | 0,00 | 5,13 |
| C1GALT1 transfers Galactose to the Tn antigen forming Core 1 glycoproteins (T antigens) | 0,00 | 5,13 |
| ST6GALNAC3/4 can add a sialic acid to the sialyl T antigen to form the disialyl T antigen | 0,00 | 5,13 |
| GCNTs transfer GlcNAc from UDP-GlcNAc to Core 1 mucins | 0,00 | 5,03 |
| ST3GAL1-4 can add a sialic acid to the T antigen at the alpha 3 position | 0,00 | 4,94 |
| Addition of GlcNAc to the Tn antigen forms a Core 3 glycoprotein | 0,00 | 4,61 |
| Termination of O-glycan biosynthesis | 0,00 | 4,54 |
| Dectin-2 family | 0,00 | 4,29 |
| GALNTs transfer GalNAc from UDP-GalNAc to mucins to form Tn antigens | 0,00 | 4,08 |
| Diseases associated with O-glycosylation of proteins | 0,00 | 3,91 |
| O-linked glycosylation of mucins | 0,00 | 3,00 |
| O-linked glycosylation | 0,00 | 2,90 |
| Diseases of glycosylation | 0,00 | 2,41 |

C. Q fever seropositives compared to healthy controls, upregulated genes

| Pathway | *P*-value | Enrichment |
| --- | --- | --- |
| Processing of Capped Intron-Containing Pre-mRNA | 0,00 | 2,87 |
| Processing of Capped Intron-Containing Pre-mRNA | 0,00 | 2,87 |
| mRNA Splicing | 0,00 | 2,62 |
| ERBB4s80:TAB2:NCOR1 complex translocates to the nucleus | 0,00 | 2,48 |
| ERBB4s80 binds TAB2:NCOR1 complex | 0,00 | 2,48 |
| ERBB4s80:TAB2:NCOR1 complex binds S100B gene promoter | 0,00 | 2,48 |
| ERBB4s80:TAB2:NCOR1 complex binds GFAP gene promoter | 0,00 | 2,48 |
| GFAP gene expression is inhibited by ERBB4:TAB2:NCOR1 | 0,00 | 2,32 |
| S100B gene expression is inhibited by ERBB4:TAB2:NCOR1 | 0,00 | 2,32 |
| Metabolism of RNA | 0,01 | 2,30 |
| Cohesin binds PDS5 and WAPAL | 0,01 | 2,27 |
| Resolution of sister chromatids | 0,01 | 2,27 |
| CDCA5 (Sororin) enables cohesion of sister chromosomal arms | 0,01 | 2,27 |
| CDCA5 (Sororin) enables cohesion of sister centromeres | 0,01 | 2,27 |
| Deacetylation of cleaved cohesin | 0,01 | 2,27 |
| Deacetylation of cohesin | 0,01 | 2,27 |
| Ankyrins link voltage-gated sodium and potassium channels to spectrin and L1 | 0,01 | 2,24 |
| mRNA Splicing - Major Pathway | 0,01 | 2,22 |
| Phosphorylation of cohesin by PLK1 at chromosomal arms | 0,01 | 2,22 |
| Acetylation of SMC3 subunit of chromosomal arm associated cohesin by ESCO1 or ESCO2 | 0,01 | 2,22 |
| Cohesin binding to decondensed chromatin is facilitated by NIPBL:MAU2 | 0,01 | 2,22 |
| Cohesin Loading onto Chromatin | 0,01 | 2,22 |
| Acetylation of SMC3 subunit of centromeric chromatin associated cohesin by ESCO1 or ESCO2 | 0,01 | 2,22 |
| Interaction between L1 and Ankyrins | 0,01 | 2,18 |
| Diseases associated with O-glycosylation of proteins | 0,01 | 2,18 |
| Signaling by ERBB4 | 0,01 | 2,17 |
| Spliceosome | 0,01 | 2,15 |
| Establishment of Sister Chromatid Cohesion | 0,01 | 2,15 |
| Lariat Formation and 5'-Splice Site Cleavage | 0,01 | 2,10 |
| CDK1 phosphorylates CDCA5 (Sororin) at chromosomal arms | 0,01 | 2,07 |
| Formation of Exon Junction Complex | 0,01 | 2,06 |

D. QFS compared to CFS, downregulated genes

| Pathway | *P*-value | Enrichment |
| --- | --- | --- |
| mRNA Splicing | 0,00 | 12,06 |
| Processing of Capped Intron-Containing Pre-mRNA | 0,00 | 11,30 |
| Processing of Capped Intron-Containing Pre-mRNA | 0,00 | 11,30 |
| mRNA Splicing - Major Pathway | 0,00 | 11,13 |
| Formation of the Spliceosomal A Complex | 0,00 | 11,03 |
| Spliceosome | 0,00 | 10,46 |
| Cleavage at the 3'-Splice Site and Exon Ligation | 0,00 | 9,57 |
| Cleavage at the 3'-Splice Site and Exon Ligation | 0,00 | 9,57 |
| Formation of the active Spliceosomal C (B*) complex | 0,00 | 9,42 |
| Formation of an intermediate Spliceosomal C (Bact) complex | 0,00 | 9,21 |
| Formation of Exon Junction Complex | 0,00 | 9,21 |
| Formation of the Spliceosomal B Complex | 0,00 | 8,85 |
| Lariat Formation and 5'-Splice Site Cleavage | 0,00 | 8,59 |
| Formation of the Spliceosomal E complex | 0,00 | 7,85 |
| mRNA Processing | 0,00 | 7,61 |
| Metabolism of RNA | 0,00 | 6,23 |
| Cleavage of Growing Transcript in the Termination Region | 0,00 | 3,76 |
| RNA Polymerase II Transcription Termination | 0,00 | 3,76 |
| Cleavage of Growing Transcript in the Termination Region | 0,00 | 3,76 |
| RNA Polymerase II Transcription Termination | 0,00 | 3,76 |
| mRNA polyadenylation | 0,00 | 3,17 |
| NXF1:NXT1 (TAP:p15) binds capped mRNA:CBC:EJC:TREX (minus DDX39B) | 0,00 | 2,79 |
| Cleavage of mRNA at the 3'-end | 0,00 | 2,72 |
| mRNA 3'-end processing | 0,00 | 2,72 |
| TREX complex binds spliced, capped mRNA:CBC:EJC cotranscriptionally | 0,00 | 2,72 |
| Systemic lupus erythematosus | 0,00 | 2,57 |
| Classical antibody-mediated complement activation | 0,00 | 2,55 |
| Activation of C1r | 0,00 | 2,55 |
| Activation of C1s | 0,00 | 2,55 |
| Formation of pre-mRNPs | 0,00 | 2,48 |
| C1-Inh binds Antigen: antibody: C1 complex activated C1r, C1s | 0,00 | 2,32 |
| Antigen:IgG:C1Q:2xActivated C1R:SERPING1:2xActivated C1S:SERPING1 dissociates | 0,00 | 2,32 |
| Chromatin modifying enzymes | 0,01 | 2,16 |
| Chromatin organization | 0,01 | 2,16 |
| SWI/SNF chromatin remodelling complex enhances MEP50:PRMT5 methyltransferase activity | 0,01 | 2,05 |
| RUNX1 binds the SWI/SNF complex | 0,01 | 2,05 |

QFS compared to CFS, upregulated genes

| Pathway | *P*-value | Enrichment |
| --- | --- | --- |
| KRAB-ZNF / KAP Interaction | 0,00 | 2,93 |
| Influenza Viral RNA Transcription and Replication | 0,00 | 2,93 |
| Nonsense-Mediated Decay (NMD) | 0,00 | 2,87 |
| p-4S-UPF1 recruits SMG5, SMG7, SMG6, PNRC2, DCP1A, and PP2A | 0,00 | 2,87 |
| Nonsense Mediated Decay (NMD) enhanced by the Exon Junction Complex (EJC) | 0,00 | 2,87 |
| Influenza Life Cycle | 0,00 | 2,82 |
| Influenza Life Cycle | 0,00 | 2,82 |
| UPF1 binds an mRNP with a termination codon preceding an Exon Junction Complex | 0,00 | 2,82 |
| SMG1 phosphorylates UPF1 (enhanced by Exon Junction Complex) | 0,00 | 2,82 |
| SMG6 hydrolyzes mRNA with premature termination codon | 0,00 | 2,79 |
| Influenza Infection | 0,00 | 2,76 |
| Influenza Infection | 0,00 | 2,76 |
| Metabolism of RNA | 0,00 | 2,74 |
| Cytoplasmic Ribosomal Proteins | 0,00 | 2,73 |
| Major pathway of rRNA processing in the nucleolus and cytosol | 0,00 | 2,56 |
| Nascent polypeptide:mRNA:ribosome complex binds signal recognition particle (SRP) | 0,00 | 2,55 |
| Translation | 0,00 | 2,55 |
| The SRP receptor binds the SRP:nascent peptide:ribosome complex | 0,00 | 2,52 |
| Formation of UPF1:eRF3 complex on mRNA with a premature termination codon and no Exon Junction Complex | 0,00 | 2,52 |
| Nonsense Mediated Decay (NMD) independent of the Exon Junction Complex (EJC) | 0,00 | 2,52 |
| Peptide chain elongation | 0,00 | 2,48 |
| Translocation of ribosome by 3 bases in the 3' direction | 0,00 | 2,48 |
| Viral mRNA Translation | 0,00 | 2,48 |
| Viral Protein Synthesis | 0,00 | 2,48 |
| Sec-tRNA(Sec):EEFSEC:GTP binds to 80S Ribosome | 0,00 | 2,48 |
| 80S:Met-tRNAi:mRNA:SECISBP2:Sec-tRNA(Sec):EEFSEC:GTP is hydrolysed to 80S:Met-tRNAi:mRNA:SECISBP2:Sec and EEFSEC:GDP by EEFSEC | 0,00 | 2,48 |
| rRNA processing in the nucleus and cytosol | 0,00 | 2,47 |
| Peptide transfer from P-site tRNA to the A-site tRNA | 0,00 | 2,47 |
| Synthesis of nascent polypeptide containing signal sequence | 0,00 | 2,47 |
| Synthesis of PB1-F2 | 0,00 | 2,47 |
| BIRC2/3 (cIAP1/2) is autoubiquitinated | 0,00 | 2,47 |
| Aminoacyl-tRNA binds to the ribosome at the A-site | 0,00 | 2,47 |
| Hydrolysis of eEF1A:GTP | 0,00 | 2,47 |
| eIF5B:GTP is hydrolyzed and released | 0,00 | 2,47 |
| The 60S subunit joins the translation initiation complex | 0,00 | 2,47 |
| Release of 40S and 60S subunits from the 80S ribosome | 0,00 | 2,47 |
| BIRC(cIAP1/2) ubiquitinates RIPK1 | 0,00 | 2,47 |
| rRNA processing | 0,00 | 2,47 |
| Regulation of necroptotic cell death | 0,00 | 2,46 |
| Polypeptide release from the eRF3-GDP:eRF1:mRNA:80S Ribosome complex | 0,00 | 2,45 |
| GTP Hydrolysis by eRF3 bound to the eRF1:mRNA:polypeptide:80S Ribosome complex | 0,00 | 2,45 |
| GTP bound eRF3:eRF1 complex binds the peptidyl tRNA:mRNA:80S Ribosome complex | 0,00 | 2,45 |
| Translation of ROBO3.2 mRNA initiates NMD | 0,00 | 2,45 |
| Translation of ROBO3.2 mRNA is negatively regulated by NMD | 0,00 | 2,45 |
| RIPK1-mediated regulated necrosis | 0,00 | 2,40 |
| Regulated Necrosis | 0,00 | 2,40 |
| Eukaryotic Translation Elongation | 0,00 | 2,39 |
| Selenocysteine synthesis | 0,00 | 2,37 |
| Eukaryotic Translation Termination | 0,00 | 2,37 |
| Recruitment of Active RNA Polymerase I to SL1:phos.UBF-1:rDNA Promoter | 0,00 | 2,31 |
| Loss of Rrn3 from RNA Polymerase I promoter escape complex | 0,00 | 2,31 |
| RNA Polymerase I Promoter Escape | 0,00 | 2,31 |
| Recruitment of Active RNA Polymerase I to SL1:phos.UBF-1:rDNA Promoter | 0,00 | 2,31 |
| Loss of Rrn3 from RNA Polymerase I promoter escape complex | 0,00 | 2,31 |
| RNA Polymerase I Promoter Escape | 0,00 | 2,31 |
| Signal-containing nascent peptide translocates to endoplasmic reticulum | 0,00 | 2,30 |
| Ribosome | 0,01 | 2,24 |
| Regulation of TNFR1 signaling | 0,01 | 2,24 |
| TNF signaling | 0,01 | 2,21 |
| TNFAIP3 (A20) ubiquitinates RIPK1 with K48-linked Ub chains | 0,01 | 2,20 |
| SRP-dependent cotranslational protein targeting to membrane | 0,01 | 2,16 |
| Formation of a pool of free 40S subunits | 0,01 | 2,12 |
| Signal peptidase hydrolyzes signal peptide from ribosome-associated nascent protein | 0,01 | 2,03 |
| NPC transports Hikeshi:HSP70s:ATP from cytosol to nucleoplasm | 0,01 | 2,02 |
| Parkin promotes the ubiquitination of mitochondrial substrates | 0,01 | 2,02 |
| RIPK1 is deubiquitinated | 0,01 | 2,01 |

**E.** QFS compared to Q fever seropositives, downregulated genes

| Pathway | *P*-value | Enrichment |
| --- | --- | --- |
| mRNA Splicing | 0,00 | 7,25 |
| Spliceosome | 0,00 | 7,17 |
| Processing of Capped Intron-Containing Pre-mRNA | 0,00 | 7,16 |
| Processing of Capped Intron-Containing Pre-mRNA | 0,00 | 7,16 |
| mRNA Splicing - Major Pathway | 0,00 | 7,03 |
| Cleavage at the 3'-Splice Site and Exon Ligation | 0,00 | 6,00 |
| Cleavage at the 3'-Splice Site and Exon Ligation | 0,00 | 6,00 |
| Formation of Exon Junction Complex | 0,00 | 5,90 |
| Formation of the Spliceosomal A Complex | 0,00 | 5,87 |
| Lariat Formation and 5'-Splice Site Cleavage | 0,00 | 5,62 |
| Formation of the active Spliceosomal C (B*) complex | 0,00 | 5,34 |
| Formation of an intermediate Spliceosomal C (Bact) complex | 0,00 | 5,18 |
| mRNA Processing | 0,00 | 4,74 |
| Chromatin modifying enzymes | 0,00 | 4,72 |
| Chromatin organization | 0,00 | 4,72 |
| Formation of the Spliceosomal B Complex | 0,00 | 4,56 |
| Circadian Clock | 0,00 | 4,21 |
| Metabolism of RNA | 0,00 | 3,65 |
| Formation of the Spliceosomal E complex | 0,00 | 3,28 |
| Cleavage of Growing Transcript in the Termination Region | 0,00 | 3,22 |
| RNA Polymerase II Transcription Termination | 0,00 | 3,22 |
| Cleavage of Growing Transcript in the Termination Region | 0,00 | 3,22 |
| RNA Polymerase II Transcription Termination | 0,00 | 3,22 |
| Circadian rythm related genes | 0,00 | 3,22 |
| Nucleolar Remodelling Complex (NoRC) binds intergenic region of rDNA | 0,00 | 2,79 |
| MTA2-NuRD complex deacetylates TP53 | 0,00 | 2,76 |
| BMAL1:CLOCK,NPAS2 activates circadian gene expression | 0,00 | 2,63 |
| SALL4 recruits NuRD to PTEN gene | 0,00 | 2,52 |
| Pathways Affected in Adenoid Cystic Carcinoma | 0,00 | 2,49 |
| NXF1:NXT1 (TAP:p15) binds capped mRNA:CBC:EJC:TREX (minus DDX39B) | 0,00 | 2,47 |
| mRNA polyadenylation | 0,00 | 2,46 |
| FKBP4 replaces FKBP5 within HSP90:ATP:FKBP5:unfolded protein | 0,00 | 2,46 |
| Cleavage of mRNA at the 3'-end | 0,00 | 2,38 |
| mRNA 3'-end processing | 0,00 | 2,38 |
| TREX complex binds spliced, capped mRNA:CBC:EJC cotranscriptionally | 0,00 | 2,38 |
| Regulation of TP53 Activity through Acetylation | 0,00 | 2,37 |
| Circadian rhythm | 0,01 | 2,28 |
| Androgen receptor signaling pathway | 0,01 | 2,25 |
| Fatty acid ligands activate PPARA | 0,01 | 2,23 |
| Fatty acid ligands activate PPARA | 0,01 | 2,23 |
| Recruitment of ERCC6 (CSB), EHMT2 (G9a), and NuRD to the promoter of rRNA gene | 0,01 | 2,23 |
| HDACs deacetylate histones | 0,01 | 2,12 |

**QFS compared to Q fever seropositives, upregulated genes**

| Pathway | *P*-value | Enrichment |
| --- | --- | --- |
| Hemostasis | 0,00 | 3,80 |
| Translation | 0,00 | 3,64 |
| Metabolism of proteins | 0,00 | 3,14 |
| SRP-dependent cotranslational protein targeting to membrane | 0,00 | 3,13 |
| Influenza Viral RNA Transcription and Replication | 0,00 | 3,09 |
| Signal peptidase hydrolyzes signal peptide from ribosome-associated nascent protein | 0,00 | 3,05 |
| Signal-containing nascent peptide translocates to endoplasmic reticulum | 0,00 | 3,02 |
| Cytoplasmic Ribosomal Proteins | 0,00 | 3,01 |
| Influenza Life Cycle | 0,00 | 2,89 |
| Metabolism | 0,00 | 2,88 |
| Influenza Infection | 0,00 | 2,66 |
| Eukaryotic Translation Elongation | 0,00 | 2,65 |
| Selenocysteine synthesis | 0,00 | 2,65 |
| Peptide transfer from P-site tRNA to the A-site tRNA | 0,00 | 2,64 |
| Synthesis of nascent polypeptide containing signal sequence | 0,00 | 2,64 |
| Synthesis of PB1-F2 | 0,00 | 2,64 |
| Ribosome | 0,00 | 2,64 |
| Peptide chain elongation | 0,00 | 2,63 |
| Translocation of ribosome by 3 bases in the 3' direction | 0,00 | 2,63 |
| Viral mRNA Translation | 0,00 | 2,63 |
| Viral Protein Synthesis | 0,00 | 2,63 |
| Aminoacyl-tRNA binds to the ribosome at the A-site | 0,00 | 2,63 |
| Hydrolysis of eEF1A:GTP | 0,00 | 2,63 |
| Sec-tRNA(Sec):EEFSEC:GTP binds to 80S Ribosome | 0,00 | 2,63 |
| 80S:Met-tRNAi:mRNA:SECISBP2:Sec-tRNA(Sec):EEFSEC:GTP is hydrolysed to 80S:Met-tRNAi:mRNA:SECISBP2:Sec and EEFSEC:GDP by EEFSEC | 0,00 | 2,63 |
| eIF5B:GTP is hydrolyzed and released | 0,00 | 2,63 |
| The 60S subunit joins the translation initiation complex | 0,00 | 2,63 |
| Release of 40S and 60S subunits from the 80S ribosome | 0,00 | 2,63 |
| Nascent polypeptide:mRNA:ribosome complex binds signal recognition particle (SRP) | 0,00 | 2,63 |
| Eukaryotic Translation Termination | 0,00 | 2,63 |
| The SRP receptor binds the SRP:nascent peptide:ribosome complex | 0,00 | 2,61 |
| Formation of UPF1:eRF3 complex on mRNA with a premature termination codon and no Exon Junction Complex | 0,00 | 2,61 |
| Nonsense Mediated Decay (NMD) independent of the Exon Junction Complex (EJC) | 0,00 | 2,61 |
| Polypeptide release from the eRF3-GDP:eRF1:mRNA:80S Ribosome complex | 0,00 | 2,60 |
| GTP Hydrolysis by eRF3 bound to the eRF1:mRNA:polypeptide:80S Ribosome complex | 0,00 | 2,60 |
| GTP bound eRF3:eRF1 complex binds the peptidyl tRNA:mRNA:80S Ribosome complex | 0,00 | 2,60 |
| UPF1 binds an mRNP with a termination codon preceding an Exon Junction Complex | 0,00 | 2,55 |
| SMG1 phosphorylates UPF1 (enhanced by Exon Junction Complex) | 0,00 | 2,55 |
| Nonsense-Mediated Decay (NMD) | 0,00 | 2,55 |
| p-4S-UPF1 recruits SMG5, SMG7, SMG6, PNRC2, DCP1A, and PP2A | 0,00 | 2,55 |
| Nonsense Mediated Decay (NMD) enhanced by the Exon Junction Complex (EJC) | 0,00 | 2,55 |
| Selenoamino acid metabolism | 0,00 | 2,48 |
| SMG6 hydrolyzes mRNA with premature termination codon | 0,00 | 2,48 |
| Mitochondrial translation | 0,00 | 2,47 |
| Mitochondrial translation elongation | 0,00 | 2,47 |
| Regulation of expression of SLITs and ROBOs | 0,00 | 2,40 |
| Major pathway of rRNA processing in the nucleolus and cytosol | 0,01 | 2,29 |
| Multi-ubiquitination of APOBEC3G | 0,01 | 2,28 |
| Formation of a pool of free 40S subunits | 0,01 | 2,28 |
| TUFM hydrolyzes GTP and TUFM:GDP dissociates from 55S ribosome | 0,01 | 2,28 |
| TUFM:GTP:aminoacyl-tRNA binds 55S ribosome:mRNA:fMet-tRNA | 0,01 | 2,28 |
| Translation of ROBO3.2 mRNA initiates NMD | 0,01 | 2,27 |
| Translation of ROBO3.2 mRNA is negatively regulated by NMD | 0,01 | 2,27 |
| Hydrolysis of GTP and dissociation of 28S and 39S subunits | 0,01 | 2,27 |
| GFM2:GTP binds 55S ribosome:mRNA:tRNA:MRRF releasing mRNA and tRNA | 0,01 | 2,27 |
| Mitochondrial translation termination | 0,01 | 2,23 |
| COMMDs displace CAND1 from cytosolic CRL E3 ubiquitin ligase complexes | 0,01 | 2,21 |
| Asparagine N-linked glycosylation | 0,01 | 2,14 |
| Peptide transfer from P-site to A-site (peptide bond formation) | 0,01 | 2,07 |
| BIRC2/3 (cIAP1/2) is autoubiquitinated | 0,01 | 2,05 |

Pathway enrichment analysis of healthy controls (n = 10), CFS patients (n = 10), QFS patients (n = 10), and asymptomatic Q fever seropositive controls (n = 10). (A) Enriched pathways when comparing CFS patients (n = 10) to healthy controls (n = 10). (B) Enriched pathways when comparing QFS patients (n = 10) to healthy controls (n = 10). (C) Enriched pathways when comparing asymptomatic Q fever seropositive controls (n = 10) to healthy controls (n = 10). (D) Enriched pathways when comparing QFS patients (n = 10) to healthy controls (n = 10). (E) Enriched pathways when comparing QFS patients (n = 10) to asymptomatic Q fever seropositive controls (n = 10).

Abbreviations: *CFS* = chronic fatigue syndrome; *QFS* = Q fever fatigue syndrome; *Q fever seropositives* = asymptomatic Q fever seropositive controls,

Enrichment scores are depicted as –Log10 of corrected *P* values.
